# Probing the Kinetic Anabolism of Poly-Beta-Hydroxybutyrate in *Cupriavidus necator* H16 Using Single-Cell Raman Spectroscopy

**DOI:** 10.3390/s16081257

**Published:** 2016-08-08

**Authors:** Zhanhua Tao, Lixin Peng, Pengfei Zhang, Yong-Qing Li, Guiwen Wang

**Affiliations:** 1 Guangxi Academy of Sciences, Nanning 530007, Guangxi, China; taozh@gxas.cn (Z.T.); penglixin@gxas.cn (L.P.); 2 Optical Imaging Laboratory at Washington University in St. Louis, One Brookings Drive, St Louis, MO 63130, USA; pengfeizhang@wustl.edu; 3 Department of Physics, East Carolina University, Greenville, NC 27858, USA; liy@ecu.edu

**Keywords:** Raman spectroscopy, laser tweezers, anabolism, poly-beta-hydroxybutyrate, single-cell analysis

## Abstract

Poly-beta-hydroxybutyrate (PHB) can be formed in large amounts in *Cupriavidus necator* and is important for the industrial production of biodegradable plastics. In this investigation, laser tweezers Raman spectroscopy (LTRS) was used to characterize dynamic changes in PHB content—as well as in the contents of other common biomolecule—in *C. necator* during batch growth at both the population and single-cell levels. PHB accumulation began in the early stages of bacterial growth, and the maximum PHB production rate occurred in the early and middle exponential phases. The active biosynthesis of DNA, RNA, and proteins occurred in the lag and early exponential phases, whereas the levels of these molecules decreased continuously during the remaining fermentation process until the minimum values were reached. The PHB content inside single cells was relatively homogenous in the middle stage of fermentation; during the late growth stage, the variation in PHB levels between cells increased. In addition, bacterial cells in various growth phases could be clearly discriminated when principle component analysis was performed on the spectral data. These results suggest that LTRS is a valuable single-cell analysis tool that can provide more comprehensive information about the physiological state of a growing microbial population.

## 1. Introduction

Plastic materials play extremely important roles in contemporary life because of their desirable properties. However, pollution caused by petroleum-based plastic waste has generated much interest in the development of more environmentally friendly substitutes, such as biodegradable polymer materials. Poly-beta-hydroxybutyrate (PHB)—a member of the polyhydroxyalkanoate family that is produced by microbial fermentation—is an attractive substitute for conventional petrochemical plastics, and has similar material properties to various thermoplastics [1]. Moreover, its excellent biodegradability, biocompatibility, piezoelectricity, and optical activity make it a widely used material in the fields of medicine, agriculture, and food production [2,3,4]. PHB is usually synthesized and accumulated in certain microorganisms as an intracellular carbon and energy storage material during times when there is a growth-limiting factor in the presence of excess carbon. The accumulated PHB is degraded and re-utilized once balanced growth conditions become available.

At present, the production cost of PHB is still much higher than that of petrochemical plastics. As a result, much effort has been devoted to making the process more economically feasible, either by finding better bacterial strains or by optimizing the PHB fermentation process [5]. *Cupriavidus necator* (formerly *Alcaligenes eutrophus* or *Ralstonia eutropha*) strain H16 is a significant PHB producer that can yield up to 80% of the dry cell weight of PHB when cultivated under suitable conditions. Its physiology, biochemistry, and genetics have been extensively studied in the context of PHB synthesis [6,7,8,9,10,11,12]. To further understand the mechanism of PHB biosynthesis in *C. necator* H16, it is very important to monitor dynamic changes in the PHB content as well as in the contents of other common biomolecules such as nucleic acids and proteins in the bacterial cells throughout the fermentation process. The conventional methods for quantifying PHB involve UV spectrophotometry [13], gas chromatography [14], or high-performance liquid chromatography [15] measurement after organic solvent extraction. However, these in vitro methods based on solvent extraction can only obtain information regarding the averaging effect of a population of cells, and mask the heterogeneity among individual cells. To better understand the regulation of PHB biosynthesis, rapid, convenient, and reliable methods for PHB quantification at the single-cell level must be developed.

Raman spectroscopy is a rapid, nondestructive, and noninvasive analytical technique that possesses multiple advantages when used in the study of biological materials [16,17,18], notably (1) water as a solvent is a weak Raman scatterer and thus has few adverse effects on Raman signals; (2) a single Raman spectrum usually contains more than 1000 Raman bands, which provide rich and intrinsic information about the molecules present in the sample (e.g., nucleic acids, proteins, carbohydrates, and lipids); (3) no or little prior sample preparation is required; and (4) a small amount of sample, even a single cell, can be analyzed. Confocal Raman spectroscopy has been used to quantify the PHB content in *C. necator* H16 [19], and to observe the content and heterogeneity of PHB in *Legionella bozemanii* [20]. However, these investigations did not focus on fluctuations in the PHB content during microorganism growth, nor did they investigate the metabolic activity of other biological components inside the bacterial cells during PHB synthesis. A particular drawback of traditional confocal Raman microscopy is that a target cell must be immobilized on a substrate using either physical or chemical approaches, and this immobilization process may change the cellular physiological state. Laser tweezers Raman spectroscopy (LTRS), which integrates optical tweezers with Raman spectroscopy, make it possible to analyze single live cells in solution [21]. LTRS has become a valuable tool for monitoring and analyzing single cell dynamics, and an overview of the LTRS method, with an emphasis on highlighting recent advances, can be found in recent review articles [22,23].

In the present study, LTRS was employed to observe the changes in the intensities of the characteristic Raman scattering peaks associated with PHB and other biomolecules during microbial batch cultivation. The results indicated that LTRS can provide comprehensive information about the metabolic activities of various biomolecules at the single-cell level and can serve as a reliable method for monitoring the PHB fermentation process.

## 2. Materials and Methods

### 2.1. Strain and Culture Medium

*Cupriavidus necator* strain H16 was used in this investigation. Nutrient agar slants contained (g/L): yeast extract 10, trypton 10, beef extract 5, NaCl 3, and agar 20, pH 7.0. The seed medium was of the same composition as the nutrient agar slants; however, the agar was eliminated. The liquid fermentation medium was composed of (g/L): fructose 20, Na_2_HPO_4_·12H_2_O 10.0, MgSO_4_·7H_2_O 0.4, (NH_4_)_2_SO_4_ 1.5, KH_2_PO_4_ 1.3, and trace element solution 1 mL, pH 7.0. The trace element solution contained (g/L): FeSO_4_·7H_2_O 10.0, ZnSO_4_·7H_2_O 2.25, CuSO_4_·5H_2_O 1.0, MnSO_4_·5H_2_O 0.5, CaCl_2_·2H_2_O 2.0, H_3_BO_3_ 0.15, Na_2_MoO_4_·2H_2_O 0.145, and 35% HCl 10 mL. Fructose was sterilized separately at 115 °C and was then reconstituted at room temperature prior to inoculation.

### 2.2. Bacterial Culture and Sampling

A loop of *C. necator* H16 cells from a nutrient agar slant was inoculated into the seed medium and cultivated at 30 °C with shaking at 200 rpm for 48 h. Then, 5% of this preculture was transferred into the fermentation culture medium and incubated at 30 °C and 200 rpm for 60 h. A 2 mL aliquot of the sample was withdrawn from the culture broth at times of 0, 3, 6, 9, 12, 15, 18, 24, 30, 36, 42, 48, 54, and 60 h. *C. necator* is a mesophile with an optimal growth temperature of 30 °C [24]. Except for certain cryophilic species, most microorganisms cease metabolic activity below about 5 °C, a temperature sometimes referred to as biological zero [25]. Hence, the above samples were kept at 4 °C until analysis.

Cell growth was monitored by measuring the absorbance of the culture broth at 600 nm using a Beckman DU800S UV/Vis spectrophotometer (Beckman Coulter, Indianapolis, IN, USA) after suitable dilution with sterile water.

### 2.3. Experimental Setup and Raman Measurements

The LTRS was set up as described by Xie and Li [21]. In brief, a circularized beam from a diode laser at 785 nm was spatially filtered and then introduced into an inverted microscope (TE2000U, Nikon, Kanagawa, Japan) equipped with an objective (×100, NA 1.30) to form a single-beam optical trap. The same laser beam was used as the excitation source. The Raman scattering light from a single cell was collected with the same objective, focused onto the entrance slit of an imaging spectrograph, and then recorded with a liquid-nitrogen-cooled (−120 °C) charge-coupled detector (Spec-10, Princeton instruments, Trenton, NJ, USA). The spectral resolution of the Raman system was approximately 6 cm^−^^1^.

A 2 μL aliquot of cold-stored culture broth was diluted 5000-fold with sterile water, and 200 μL of this cell suspension was loaded into the hole of a sample holder. A single bacterial cell was captured by the focused laser beam and levitated 5 μm above the quartz cover slip to reduce the florescence background from the substrate. The Raman measurement of an individual cell was performed using a laser power of 18 mW and an exposure time of 120 s. The spectra of 30 randomly selected cells were recorded for each cell suspension. The aforementioned procedure for the suspension preparation and the Raman measurement was repeated five times—i.e., a total of 150 spectra were collected for each time point. For calibration, pure PHB particles suspended in sterile water were trapped with laser tweezers, and their Raman spectra were recorded using an exposure time of 10 s.

### 2.4. Spectral Data Analysis

The raw spectral data were saved in ASCII format and imported into Origin 6.0 (OriginLab Corporation, Northampton, MA, USA) for further data processing. The background subtraction and response function calibration were conducted as follows: *S*_act_(*v*) = (*S*_acq_(*v*) − *S*_bg_(*v*))/*R*(*v*), where *S*_act_(*v*) is the actual spectrum of a single cell, *S*_bg_(*v*) is the background spectrum, *S*_acq_(*v*) is the raw spectrum acquired by LTRS, and *R*(*v*) is the response function of the instrument. The intensities of individual characteristic bands were calculated using a program written in Matlab 6.5 (Mathworks, Inc., Natick, MA, USA). The spectral region containing the most relevant information (650–1800 cm^−1^) was used for a principal component analysis (PCA). The PCA was performed using Unscrambler 9.7 (Camo, Oslo, Norway) after the raw spectral data were smoothed using a three-point adjacent-averaging filter, background-subtracted, and baseline-corrected.

## 3. Results and Discussion

### 3.1. Raman Spectra of Single *C. necator* Cells

**Figure 1:**
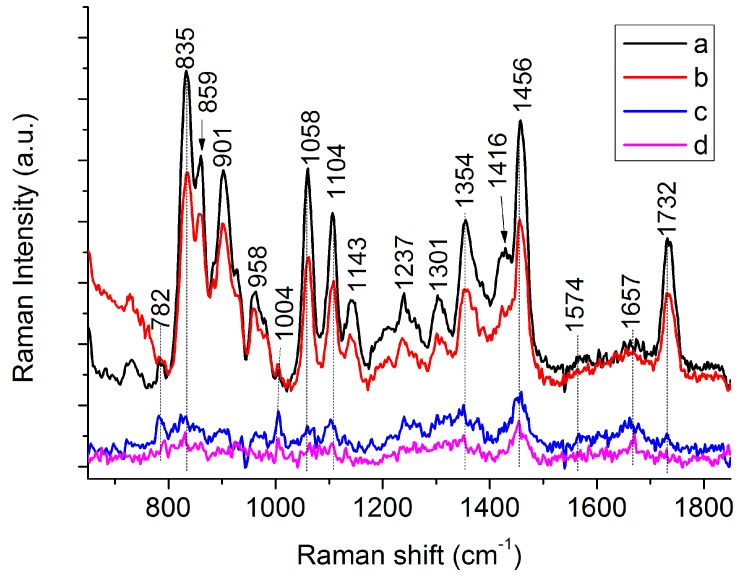
Single-cell Raman spectra from *Cupriavidus necator* H16 cells cultured for 24 h (a,b) or 2 h (c,d). Spectra were acquired from cells while trapped (a,c) or adhered onto quartz cover (b,d) by laser tweezers Raman spectroscopy (LTRS). Curves c and d were amplified by factor of two.

Trapping a bacterial cell above a slide with laser tweezers not only allows the acquisition of Raman scattering from the entire cell and analysis of the chemical composition within the cell, but also greatly reduces the background level resulting from scattering from the slide. A comparison between the Raman spectra of an individual cell first captured in suspension and subsequently attached onto the quartz slide is shown in Figure 1. This figure clearly shows that a stronger Raman signal and higher signal-to-noise ratio can be obtained if the cell is levitated 5 μm above the quartz cover slip.

**Figure 2:**
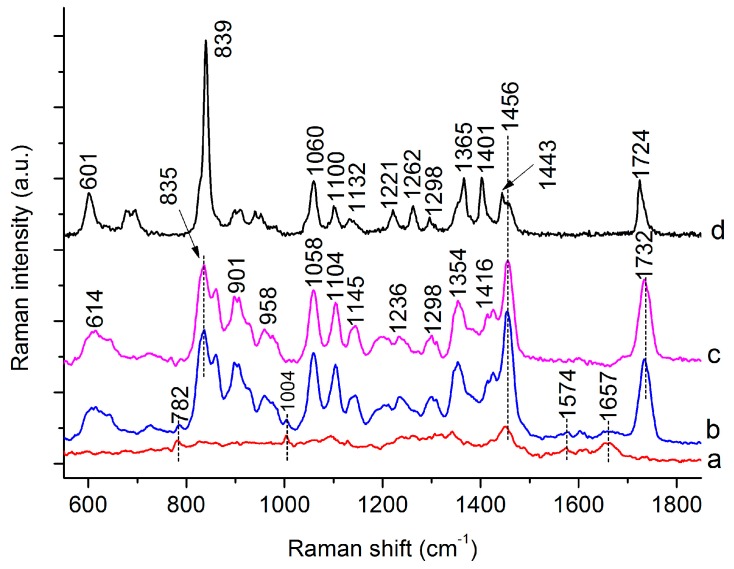
Raman spectra of H16 cells cultured for 0 h (curve a) and 48 h (curve b), the differential spectrum between curve b and a (curve c), and reference Raman spectrum of poly-beta-hydroxybutyrate (PHB) standard (curve d).

To verify the accumulation of PHB in the *C. necator* cells during batch culture, the Raman spectra of commercially available PHB, as well as those of bacterial cells cultivated for 0 and 48 h, were recorded (Figure 2, curve a,b). A comparison of the differential spectrum (Figure 2, curve c) obtained from the Raman spectra of *C. necator* cells at 48 and 0 h with the reference spectrum of pure PHB (Figure 2, curve d) shows that Raman scattering originating from PHB dominates the differential spectra, confirming PHB production during the cultivation period of 0–48 h. Major characteristic bands specific to PHB in *C. necator* cells are located at 835, 901, 1058, 1104, 1354, 1456, and 1732 cm^−1^. Some Raman peaks are shifted compared with those of the reference spectrum, which may result from differences in the physical state of PHB, with the amorphous state present in bacterial cells and the crystalline state in pure PHB [26]. As shown in Figure 1, the characteristic band at 1732 cm^−1^ is very strong, and there are no other peaks nearby. Therefore, this peak was chosen to quantify the PHB content in the *C. necator* cells. Apart from the bands originating from PHB, bands associated with nucleic acids (located at 782 and 1574 cm^−1^) and proteins (located at 1004 and 1657 cm^−1^) are observable in the Raman spectra of the *C. necator* cells (Figure 2, curve b). These bands could provide abundant information regarding the composition and structure of intracellular molecules in individual bacterial cells. Tentative assignments for the Raman peaks of the PHB and H16 cells are summarized in Table 1.

### 3.2. Dynamic Changes in Biomolecule Level inside *C. necator* H16 Cells during Batch Culture

**Figure 3:**
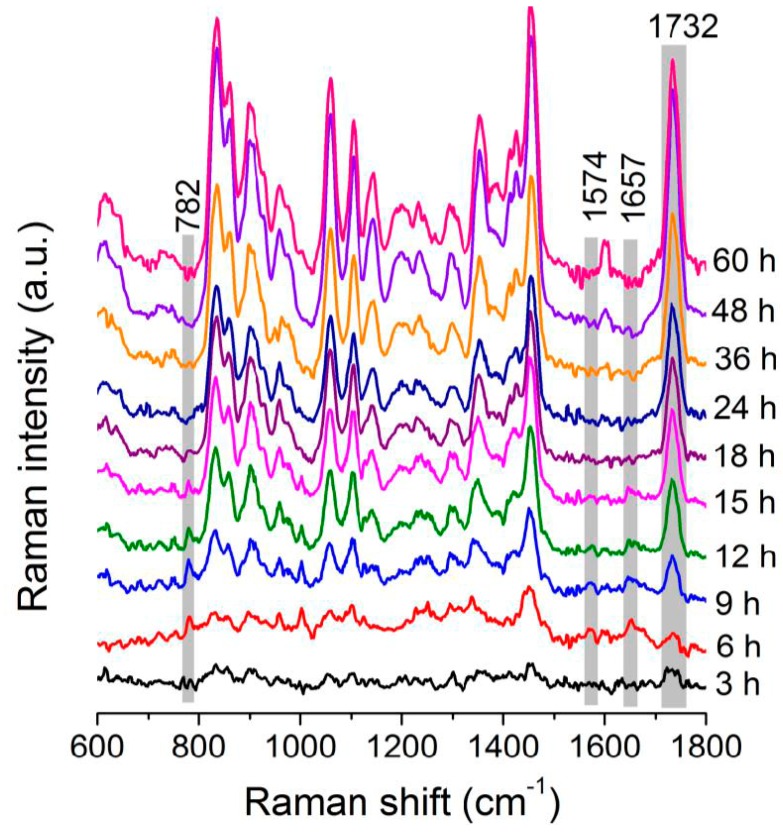
Averaged Raman spectra of single *C. necator* H16 cells, taken at different incubation times, after subtraction from averaged spectra taken at 0 h.

The time-lapse differential spectra between the Raman spectra of cellular samples taken at various time points and those taken at 0 h are shown in Figure 3; these spectra provide information about the changes in the components of the *C. necator* H16 cells during fermentation. A significant increase in the intensities of the Raman bands assigned to PHB can be observed, indicating that PHB continuously accumulated inside the cells during this period. Changes in the Raman scattering of other biomolecules present in the bacterial cells also contributed to the sequential differential spectra. To fully understand the substance metabolism that occurs during the course of PHB fermentation, the fluctuation in the intensities of the Raman bands originating from various biomolecules in the cells was investigated in detail.

**Figure 4:**
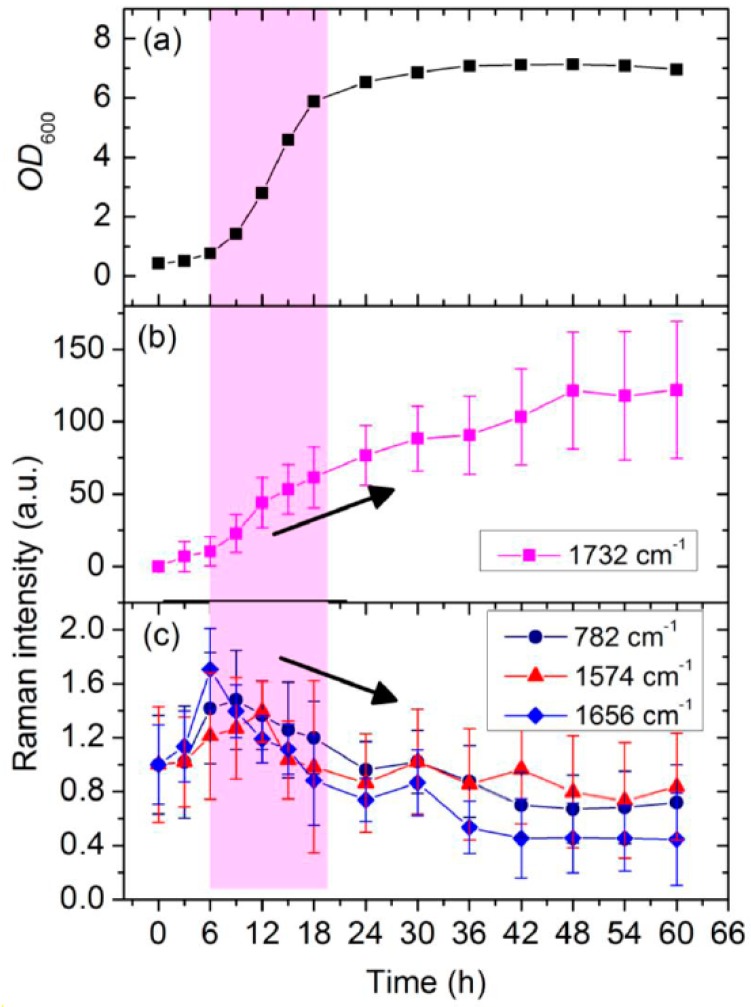
(**a**) Growth curve of a single culture of *C. necator* H16; (**b**) kinetic intensities of peaks at 1732 cm^−1^ (PHB); and (**c**) kinetic intensities of peaks at 782 cm^−1^ (RNA), 1574 cm^−1^ (DNA), and 1656 cm^−1^ (proteins), as functions of incubation time. The pink bar illustrates exponential phase of cell growth, and the error bar is standard deviation.

The intensities of the Raman bands at 782, 1575, 1656, and 1732 cm^−1^ were used to determine the contents of RNA, DNA, proteins, and PHB, respectively. Figure 4 presents the cellular growth curve, as well as the time-dependent Raman intensities of these molecules during PHB fermentation. As shown in Figure 4a,b, PHB accumulation began in the early stage—i.e., during the lag phase (3 h)—of the batch culture of *C. necator*. The growth curve shows normal cellular growth behavior and thus sufficient nutrient supply; therefore, nutrition limitation does not appear to be the trigger for PHB synthesis in this strain. The maximum PHB production rate is observable in the early and middle exponential phases (6–18 h). The highest intracellular PHB level is evident after 48 h; following this time, the PHB Raman signal diminishes slowly because some PHB was re-utilized as a carbon source. This description of time-dependent changes in PHB content inside cells of *C. necator* during batch growth is consistent with that measured by Shilpi Khanna [30] using gas chromatography. These results show that PHB production occurred during most periods of *C. necator* H16 batch growth, which may be because PHB polymerization is a relatively simple chemical process, with simple requirements in terms of the substrates and enzymes needed [31].

RNA, DNA, and proteins are common biomolecules within cells, and can be seen as markers for metabolic activity involved in cellular growth. Monitoring the changes in the Raman signals related to these substances helps to understand the cellular physiological state and the regulation of PHB biosynthesis during fermentation. The intensities of the Raman peaks specific to RNA and proteins (782 and 1656 cm^−1^, respectively) behaved similarly (Figure 4c)—i.e., a drastic increase occurs soon after inoculation, reaching the maximum during the early exponential phase (6–9 h), and slowly declining thereafter. The intensities drop to their initial levels at the end of the late exponential phase and decrease continuously until the minimum values (45%–70% of the initial levels) are finally attained sometime during the stationary phase. The active biosynthesis of RNA and proteins in bacterial cells during the lag phase may be a response to the environmental stress experienced when the microbial culture was transferred from the seed culture medium to the fermentation medium. Upon a medium downshift, the cells must transcribe and translate the genes encoding the enzymes participating in the syntheses of the essential metabolites that are not present in the fresh medium. Hence, the amounts of RNA and proteins associated with this process increase during the lag phase. The intensity of the DNA peak (at 1574 cm^−1^) rises during the early exponential phase (6–12 h) and then decreases throughout the remaining cultivation period. During the early exponential phase, most of the *C. necator* cells are in rapid proliferation, whereas during the late exponential and stationary phases, they are in growth arrest. Cells that are proliferating have more DNA than those whose growth is arrested as a result of chromosomal DNA replication [32].

### 3.3. Analysis of the Heterogeneity of PHB Production at the Single-Cell Level

**Figure 5:**
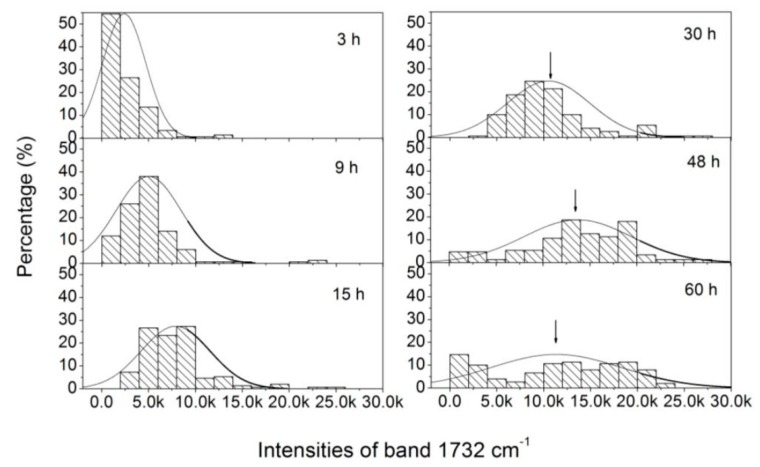
Histograms of intensities of Raman band at 1732 cm^−1^, obtained from 150 *C. necator* H16 cells after 3, 9, 15, 30, 48, and 60 h of incubation.

In genetically homogeneous microbial cultures, single cells deviate from each other in terms of life cycle. This cellular heterogeneity implies that, to understand cellular behavior, it is important to study how individual cells respond to environmental signals. In this work, variations in the level of PHB per cell were assessed over 150 single cells of *C. necator* H16. Samples were taken at different times (3, 9, 15, 30, 48, and 60 h), and Figure 5 shows the histograms obtained for the Raman band intensities of the peak at 1732 cm^−1^ at these times. During the lag phase (3 h), no or extremely weak PHB Raman scattering is observable in more than 50% of the bacterial cells, whereas the PHB signal is detectable in more than 80% of the cells during the early exponential phase (9 h). When the fermentation enters the middle exponential phase (15 h) and the stationary phase (30 h), PHB Raman scattering can be observed in all of the cells. The PHB content inside individual cells is relatively homogenous and exhibits an approximately normal distribution within the bacterial cultures. In the late stage of the fermentation (48 and 60 h), PHB Raman scattering among individual cells within the cellular populations becomes very inhomogeneous and exhibits a very broad distribution (ranging from 0 to 3 × 10^4^ counts). After 60 h, approximately 15% of the cells exhibit no PHB signals, implying that some cells had begun to degrade and utilize PHB in response to the lack of carbon in the culture medium. The results presented here show that LTRS method can assess the heterogeneity within a *C. necator* cell population under dynamic process conditions. Integrating such information with microbial population characteristics measured via conventional analysis methods may contribute significantly to understanding the physiology of a growing microbial population, and should provide detailed data useful for the evaluation, design, and control of PHB fermentation processes.

### 3.4. PCA of Raman Spectra of Individual *C. necator* Cells at Different Growth Stages

**Figure 6:**
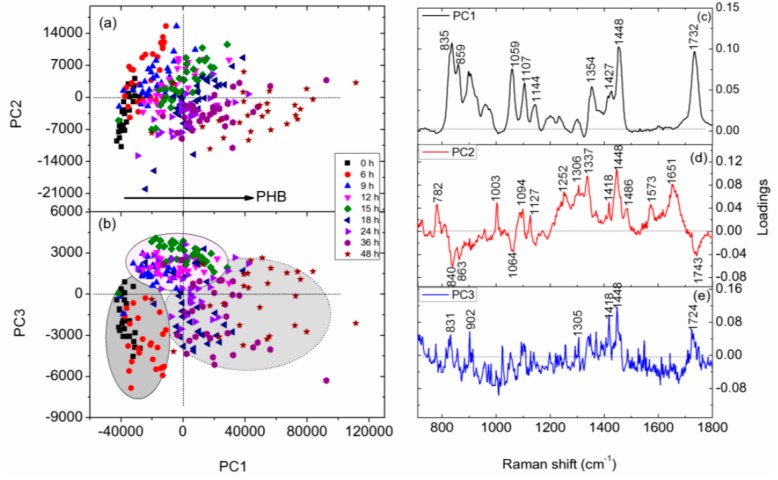
Principal components analysis (PCA) of Raman spectra of individual H16 cells at different culture time. (**a**,**b**) Scatter plots of PCA scores; (**c**–**e**) loadings of PC1, PC2, and PC3, respectively.

PCA is a sensitive chemometric technique that can recognize small spectral variations in large sets of Raman spectroscopic data. In this investigation, PCA was performed on the Raman spectra of *C. necator* H16 cells cultivated for 0, 6, 9, 15, 18, 24, 36, and 48 h (Figure 6). In the scatter plot of PC1 and PC3 scores (Figure 6b), the spectral data for bacterial cells are clustered into three groups according to the microbial growth phase: 0 and 6 h, corresponding to the lag phase; 9, 12, and 15 h, corresponding to the exponential phase; and 18, 24, 36, and 48 h, corresponding to the stationary phase. PC loadings can provide insight into the physical basis responsible for this discrimination. The relatively high loadings on PC1 and PC3 are Raman bands assigned to PHB at 835, 901, 1058, 1104, 1354, 1418, 1456, and 1732 cm^−1^ (Figure 6c,e), indicating that the variations in PHB content inside *C. necator* H16 cells at different growth stages contributed mostly to the separation observed in the above PCA scatter plot. By contrast, Raman bands associated with nucleic acids (located at 782, 1094, 1337, 1486, and 1573 cm^−1^) and proteins (located at 1003, 1448, and 1651 cm^−1^) dominate the PC2 loading plot (Figure 6d); hence, the PC2 score can be seen as a measure of bacterial growth activity—i.e., the larger the PC2 score value, the more active the bacterial growth. Examination of the scatter plot of PC1 and PC2 scores (Figure 6a) allows identification of the metabolic states of *C. necator* H16 cells in various cultivation stages. Most bacterial cells in the lag phase and early and middle exponential phases (3–15 h)—which exhibit high PC2 score values but low PC1 score values—were in the active growth state, whereas the bacterial cells in the stationary phase—with low PC2 score values but high PC1 score values—accumulated large amounts of PHB and were in the growth arrest state.

## 4. Conclusions

The present study demonstrated the use of LTRS for monitoring dynamic changes in the contents of PHB and of other common biomolecules inside *C. necator* H16 cells during batch growth at both the population and single-cell levels. PHB accumulation began in the early stage of the batch culture, and the maximum PHB production rate was observed in the early and middle exponential phases. The active biosynthesis of DNA, RNA, and proteins occurred during the lag and early exponential phases, whereas the levels of these biomolecules decreased continuously in the middle, late exponential, and stationary phases, until the minimum values were obtained (45%–70% of initial levels). LTRS can also provide novel insights into the heterogeneity of the PHB content inside individual cells within the cellular population at certain time points during the batch culture. The PHB content inside individual cells was relatively homogenous within the cellular populations during the middle stage of fermentation, while the variation in PHB levels among individual cells within bacterial cultures during late stages of microbial growth increased. In addition, bacterial cells in the lag, exponential, and stationary phases could be clearly identified and visualized by performing PCA on the spectral data. The variation in PHB content inside *C. necator* H16 cells contributes mostly to the separation observed in the PCA scatter plots. These results suggest that LTRS is a valuable single-cell analysis tool that can provide more comprehensive information about the physiological state of a growing microbial population.

## Figures and Tables

**Table 1:** Tentative assignments for Raman peaks of PHB and H16 cells.

Peaks (cm^−1^)			Assignment ^a^
0 h Cell	24 h Cell	PHB	
782	782		G, U of RNA [19,27]
832	835	839	C-O-C str. of PHB [26,28], “exposed” Tyr of cells [29]
	859	859	C-O-C str. of PHB [28], “buried” Tyr of cells [27,29]
	901	899	*υ*(COC) [19]
	958	952	C-C str. and CH_3_ rocking of PHB [26,28]
1004	1004		Phe [27,29]
	1058	1059	C-O str. [26,28]
1094			PO^2-^ str. of DNA [27,29]
	1104	1100	C-O-C sym. str. of PHB [26,28]
1129	1143	1132	1129, C-N str. of proteins [27,29]
			1143, (C-N), (C-C) str. of proteins [27,29]
			1132, C-O-C sym. str. of PHB [26,28]
	1237	1221	1237, amide III [19,27]
			1221, C-O-C asymmetric str. [26,28]
1262	1262	1262	C-O-C str. and CH def. [26,28]
			Helical conformation (C) of PHB [26,28]
1305	1301	1294	CH_2_ twist of lipids, CH def. of PHB [26,28]
1342			A, G of nucleic acid and C–H of proteins. [19,27]
	1354	1365	Sym. str. CH_3_ and CH def of PHB [26,28]
	1416	1401	CH_3_ sym. def. [26,28]
1453	1456	1443	CH_2_/CH_3_ [26,28]
1574	1574		G, A of DNA [19,27]
1657	1657		Amide I [19,27]
	1732	1724	1732 C=O str. of PHB (Amorphous) [26]/C=O str. of lipids [29]
			1724 C=O str. of PHB (Crystalline) [26]

^a^ str: stretching; def: deformation; sym: symmetric.
